# Value of Chest Radiographic Pattern in RSV Disease of the Newborn: A Multicenter Retrospective Cohort Study

**DOI:** 10.1155/2012/861867

**Published:** 2012-12-10

**Authors:** Américo Gonçalves, Gustavo Rocha, Hercília Guimarães, Paula Cristina Fernandes, Elisa Proença, Dulce Oliveira, Paula Rocha, Conceição Quintas, Teresa Martins, Alice Freitas, Clara Paz Dias, Albina Ramires

**Affiliations:** ^1^Division of Neonatology, São João Hospital, Alameda Prof. Hernâni Monteiro, 4200-319 Porto, Portugal; ^2^Division of Pediatric and Neonatal Intensive Care, Santo António General Hospital, 4099-001 Porto, Portugal; ^3^Unit of Neonatology, Júlio Dinis Maternity, 4050-371 Porto, Portugal; ^4^Unit of Neonatology, M**ª**Pia Children's Hospital, 4050-311 Porto, Portugal; ^5^Unit of Neonatology, Vila Nova de Gaia Hospital, 4434-502 Vila Nova de Gaia, Portugal; ^6^Unit of Neonatology, Pedro Hispano Hospital, 4454-509 Matosinhos, Portugal; ^7^Unit of Neonatology, Senhora da Oliveira Hospital, 4835-044 Guimarães, Portugal; ^8^Division of Neonatology, Braga Hospital, 4710-243 Braga, Portugal

## Abstract

Respiratory syncytial virus (RSV) lower respiratory tract infection is the most common viral respiratory infection in infants. Several authors have sought to determine which risk factors are the best predictors for severe RSV disease. Our aim was to evaluate if a specific chest radiographic pattern in RSV disease can predict the disease severity. We conducted a multicenter retrospective cohort study in term and preterm neonates with confirmed lower respiratory tract RSV infection, admitted to neonatal intensive care units (NICU) from 2000 to 2010. To determine which factors independently predicted the outcomes, multivariate logistic regression analysis was performed. A total of 259 term and preterm neonates were enrolled. Patients with a consolidation pattern on the chest radiograph at admission (*n* = 101) had greater need for invasive mechanical ventilation (OR: 2.5; *P* = .015), respiratory support (OR: 2.3; *P* = .005), supplemental oxygen (OR: 3.0; *P* = .008), and prolonged stay in the NICU (>7 days) (OR: 1.8; *P* = .025). Newborns with a consolidation pattern on admission chest radiograph had a more severe disease course, with greater risk of invasive mechanical ventilation, respiratory support, supplemental oxygen, and prolonged hospitalization.

## 1. Introduction

Respiratory syncytial virus (RSV) lower respiratory tract infection is the most common viral respiratory infection in infants [1]. It is characterized by acute inflammation, edema, and necrosis of epithelial cells lining small airways, increased mucus production, and bronchospasm. Radiographically, RSV lower respiratory tract infection can present itself predominantly as bronchiolitis, a pulmonary obstructive disease with hyperinflation, or as pneumonitis, a restrictive parenchymal disease with diffuse consolidation areas [2, 3]. Although neonatal RSV infection is relatively infrequent [4], newborns have a significant risk for severe disease (i.e., need for mechanical ventilation and/or death) [5]. The ability to predict which neonates will have a more severe disease course could help in the selection of treatment facilities and guide management strategies. Several authors have sought to determine which risk factors are the best predictors for severe RSV disease. Younger age at presentation, lower birthweight, prematurity, congenital heart disease, chronic lung disease, and immunodeficiency have consistently been associated with greater chance for hospital admission, longer hospital stay, and need for mechanical ventilation in RSV-infected infants [6–11].

Several studies have sought to determine which radiographic findings are more frequently associated with RSV infection [12–14]. Some authors have suggested that specific chest radiographic patterns in RSV-infected infants were related with disease course and severity [6, 15–17]. 

Our aim was to evaluate if a specific chest radiographic pattern (consolidation) in RSV infection can independently predict disease severity, namely, the need for supplemental oxygen, respiratory support, invasive mechanical ventilation, and prolonged length of hospitalization, in a newborn population.

## 2. Material and Methods

### 2.1. Study Design and Population

In order to establish the relative importance of chest radiographic patterns in RSV disease of the newborn, we conducted a multicenter retrospective cohort study, spanning an eleven-year period (2000–2010) by abstracting relevant data from clinical charts and birth files in eight level III-Neonatal intensive care units (NICU). 

Term and preterm neonates, (≤28 days of life and/or ≤44 weeks corrected gestational age at time of diagnosis), with confirmed lower respiratory tract RSV infection (positive detection of viral RNA in respiratory secretions), admitted to a NICU were included.

The institutional ethics committee approval was obtained in all participant institutions.

### 2.2. Data Collection

Medical records were reviewed for (1) RSV diagnosis confirmed by viral diagnostic testing; (2) demographic characteristics including gender, birth gestational age, birthweigth, and corrected gestational age at time of diagnosis; (3) underlying medical conditions such as prematurity, congenital heart disease, and bronchopulmonary dysplasia (according to the National Institute of Health Consensus); (4) disease severity markers including length of stay in the NICU, need and duration of respiratory support (invasive mechanical ventilation (IMV) and/or continuous positive airway pressure (CPAP)), and requirement for supplemental oxygen therapy; (5) development of complications including pneumothorax, bacterial pneumonia, sepsis, and death; (6) chest radiographic findings grouped in two different categories: consolidation versus hyperinflation. 

### 2.3. Chest Radiographic Patterns

**Figure 1:**
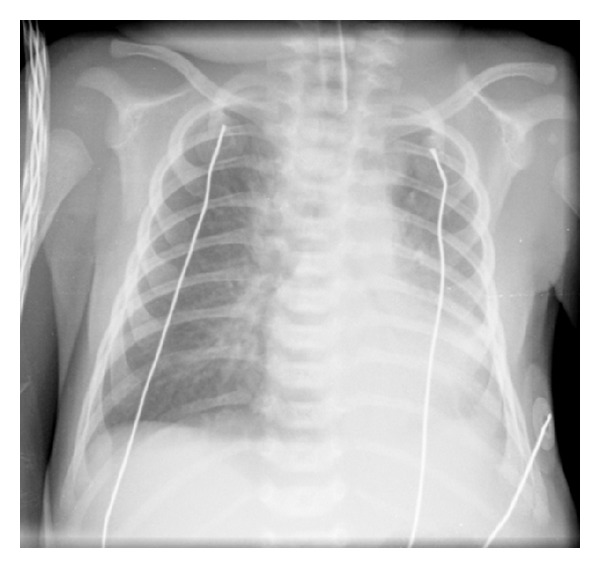
Consolidation pattern-chest radiograph showing pulmonary lower left lobe consolidation.

**Figure 2:**
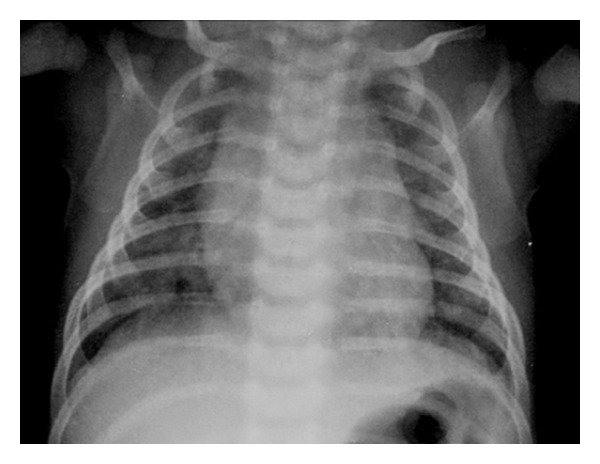
Hyperinflation pattern-chest radiograph showing pulmonary bilateral hyperinflation.

Patients with alveolar infiltrates and/or opacities with bronchogram (“white lung”) were considered as having a consolidation pattern (Figure 1). Patients with hyperinflated or normal radiograph (“black lung”) were considered as having a hyperinflation pattern (Figure 2). Chest radiographic characterization was based on a chest radiograph taken within the first 24 hours after admission. When multiple chest radiographs were taken, the one with the most significant radiological findings was considered. Patients whose radiographs could not be clearly classified within those two categories, or had been taken ≥24 h after admission, were excluded from the analysis.

### 2.4. Statistical Analysis

Descriptive statistics of patient characteristics were performed and reported in terms of mean and standard deviation (SD) for the quantitative variables and absolute frequencies and percentages for the qualitative variables.

Demographic characteristics and risk factors were subjected to univariate analysis using the *χ* ^2^ test or Fisher's exact test for categorical variables and a 2-tailed Student's *t*-test or Mann-Whitney test for continuous variables, as appropriate. 

As markers of severe disease, we selected the following primary outcomes: need for respiratory support need for invasive mechanical ventilation; supplemental oxygen requirement; length of stay in the NICU (dichotomized at >7 days using the median value of the variable).

To determine which factors independently predicted the outcomes, statistical models were built by using multivariate logistic regression analysis (backward stepwise). Variables statistically significant in the univariate analysis and/or considered clinically relevant for the outcome were entered in the model. Six potential independent predictors were considered: birthweight, gender, prematurity, chest radiographic pattern, congenital heart disease (CHD) and bronchopulmonary dysplasia (BPD).

Association of predictors with the primary outcomes was displayed using odds ratios (OR) and 95% CI's. Predictor variables with a *P-*value of <0.05 and multivariate odds ratios (and 95% CI's) that did not include 1 were considered significant. Statistical analyses were performed by using PASW statistics 18.0.

## 3. Results

From the 273 patients who met the inclusion criteria 14 (5.1%) were excluded: 12 (4.4%), due to inability to clearly classify the chest radiograph and 2 (0.7%) due to missing data. Of the 259 remaining patients, 139 (53.7%) were male. The mean (±SD) gestational age was 37.3 ± 2.8 weeks, with 71 (27.4%) being preterm infants (<37 weeks). Corrected gestational age at time of diagnosis was 40.1 ± 2.3 weeks and the mean (±SD) birthweight was 2921 ± 692 grams. 

### 3.1. Univariate Analysis

First we compared the baseline demographic characteristics and risk factors between patients with a consolidation pattern on chest radiography (CPCR) versus patients with a hyperinflation pattern on chest radiography (HPCR). Patients with CPCR were predominantly females and had lower birth and corrected gestational age. No significant differences were found between groups in any of the studied risk factors (Table 1).

Secondly, we compared differences in disease severity markers. The proportion of infants who required supplemental oxygen therapy, respiratory support, and invasive mechanical ventilation was significantly higher in the CPCR group with significantly longer median length of stay in the NICU and duration of supplemental oxygen therapy (Table 2).

Finally, we observed that CPCR patients were significantly more prone to develop complications with three times more cases of bacterial pneumonia when compared to HPCR patients (Table 3).

### 3.2. Multivariate Analysis

**Figure 3:**
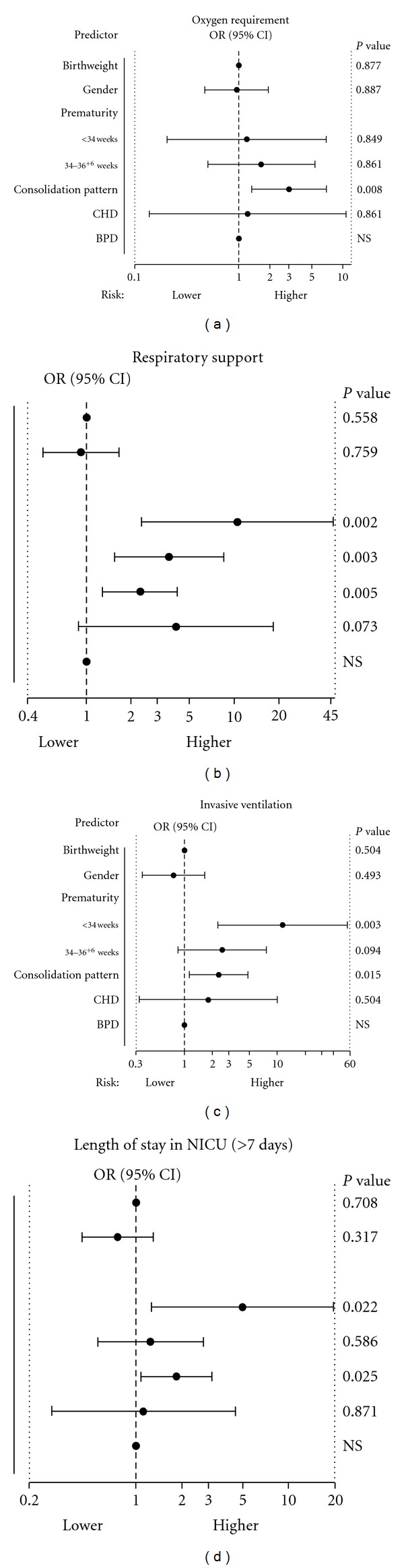
Odds ratios (ORs) for risk factors associated with disease severity in newborns with respiratory syncytial virus hospitalized in neonatal intensive care. According to multiple logistic regression analyses, the independent significant risk factors associated with disease severity, that is length of stay in NICU (≤7 versus >7 days), requirement of oxygen, respiratory support and invasive ventilation are those with a *P* value of <0.05. The reference category for gender is male, for prematurity (<34 and 34–36^+6^ weeks) is term (≥37 weeks) and for consolidation pattern is hyperinflation. NS indicates not significant.

Of the considered predictors, prematurity (particularly in infants <34 weeks of gestational age) and chest radiograph pattern were independently associated with the need for respiratory support, need for invasive mechanical ventilation, and length of stay in the NICU > 7 days. Only the chest radiographic pattern was found to be an independent predictor for all four markers of disease severity (Figure 3).

## 4. Discussion

Several risk factors have been used to predict severe disease in RSV infected infants. Younger age at presentation, lower birthweight, prematurity, congenital heart disease, chronic lung disease, and immunodeficiency have consistently been associated with greater chance for hospital admission, longer hospital stay, and need for mechanical ventilation in RSV-infected infants [6–11]. However, currently existing models still fail to predict disease evolution in a considerable number of patients, suggesting that there are additional factors yet to be considered in risk stratification. 

Our study showed that newborns with a consolidation chest pattern had more severe disease with greater need for supplemental oxygen, respiratory support, invasive mechanical ventilation, and longer length of stay in the NICU. These observations support the relevance of chest radiographic pattern in RSV-infected newborns, as had been previously suggested [6, 15–17]. Indeed, it could serve as a surrogate marker of lower respiratory tract disease pattern in RSV disease. Prematurity (particularly those ≤34 weeks of gestational age) was also found to be an independent risk factor for severe disease in our population, with none of the other risk factors showing an independent effect. 

Our study had a few limitations. Firstly, the sample size allowed only the detection of risk factors strongly associated with the primary outcomes and that were prevalent in our study population. The small numbers of congenital heart disease and bronchopulmonary dysplasia present in our sample could have underestimated their effect and results must be interpreted with caution. Low bronchopulmonary dysplasia (diagnosed according to the National Health Institute Consensus criteria) [18] prevalence relates to the low number of very premature newborns (<32 weeks) with RSV infection, which in turn could be explained by the universal use of anti-RSV human recombinant monoclonal antibody (palivizumab) in those patients [19].

Secondly, some patients cannot be clearly classified has having a consolidation or a hyperinflation pattern based on admission chest radiographs. Although such classification is possible in the vast majority of patients, some will have incipient or equivocal findings requiring chest radiograph repetition at a later time which falls beyond the scope of our study. 

This study focused on a newborn population, for which there are few available data. We have shown that a consolidation pattern in RSV disease of the newborn is an independent predictor of disease severity and should be considered in clinical prediction rules. Better prediction of disease severity risk on admission will allow differential management strategies and more adequate resource allocation. 

## 5. Conclusion

RSV-infected newborns with low gestational age (particularly those ≤34 weeks) and a consolidation pattern on admission (first 24 h) should be considered as high risk patients for a severe disease course, with greater risk of invasive mechanical ventilation, respiratory support, supplemental oxygen, and prolonged hospitalization.

## Figures and Tables

**Table 1:** Demographic characteristics and risk factors of newborns hospitalized in NICU with an RSV infection presenting a CPCR versus HPCR pattern.

	CPCR (*N* = 101)	HPCR (*N* = 158)	*P*
Demographic characteristics			
Gestational age, mean ± SD, wk	36.3 ± 3.0	37.6 ± 2.6	0.012^a^
Weight, mean ± SD, g	2828 ± 744	2980 ± 653	0.085
Gender, *n* (%)			0.036^a^
Male	46 (45.5)	93 (58.9)	
Female	55 (54.5)	65 (41.1)	
Corrected gestational age, mean ± SD, wk	39.6 ± 2.3	40.4 ± 2.2	0.006^a^
Postnatal infection, mean ± SD, d	23.7 ± 13.3	21.8 ± 14.2	0.285
Risk factors			
Prematurity, *n* (%)	33 (32.7)	38 (24.1)	0.129
Gestational age < 34 wk	12 (11.9)	12 (7.6)	
Gestational age 34–36^+6^ wk	21 (20.8)	26 (16.5)	
Congenital heart disease, *n* (%)	6 (5.9)	3 (1.9)	0.083
Bronchopulmonary dysplasia, *n* (%)	2 (2.0)	0 (0.0)	0.151

NICU: neonatal intensive care unit; RSV: respiratory syncytial virus; CPCR: consolidation pattern in chest radiography; HPCR: hyperinflation pattern in chest radiography; ^a^significant differences.

**Table 2:** Disease characteristics in infants hospitalized in NICU with an RSV newborns presenting a CPCR versus HPCR pattern.

Markers of disease severity	CPCR (*N* = 101)	HPCR (*N* = 158)	*P*
Length of stay, median (IQR (25th–75th percentile)), d	8 (5–12)	7 (4–9)	0.005^a^
Supplemental oxygen			
Requirement, *n* (%)	93 (92.0)	124 (78.5)	0.004^a^
Duration, median (IQR (25th-75th percentile)), d	4 (3–7)	3 (2–5)	0.003^a^
O_2_ maximum concentration, median (IQR (25th–75th percentile)), (%)	30 (28–50)	30 (27–38)	0.085
Respiratory support			
Requirement, *n* (%)	50 (49.5)	46 (29.1)	0.001^a^
Duration, median (IQR (25th–75th percentile)), d	3 (1–5)	2 (1–3)	0.184
Invasive mechanical ventilation			
Requirement, *n* (%)	23 (22.8)	17 (10.8)	0.009^a^
Duration, median (IQR (25th–75th percentile)), d	4 (2–6)	3 (1–4)	0.137
Maximum inspiratory pressure, median (IQR (25th–75th percentile)), mmHg	22 (20–28)	20 (20–23)	0.257

NICU: neonatal intensive care unit; RSV: respiratory syncytial virus; CPCR: consolidation pattern in chest radiography; HPCR: hyperinflation pattern in chest radiography; IQR: interquartile range; ^a^significant differences.

**Table 3:** Complications developed in newborns hospitalized in NICU with a RSV infection presenting a CPCR versus HPCR pattern.

	CPCR (*N* = 101)	HPCR (*N* = 158)	*P*
Pneumothorax, *n* (%)	0 (0)	1 (0.63)	0.610
Pneumonia, *n* (%)	42 (41.6)	21 (13.3)	<0.001^a^
Sepsis, *n* (%)	2 (2.0)	2 (1.3)	0.649
Death, *n* (%)	1 (1.0)	0 (0)	0.391

NICU: neonatal intensive care unit; RSV: respiratory syncytial virus; CPCR: consolidation pattern in chest radiography; HPCR: hyperinflation pattern in chest radiography; ^a^Significant differences.

## References

[1] Leader S, Kohlhase K, Pearlman MH, Williams JV, Engle WA (2003). Recent trends in severe respiratory syncytial virus (RSV) among US infants, 1997 to 2000. *Journal of Pediatrics*.

[2] Greenough A (2009). Role of ventilation in RSV disease: CPAP, ventilation, HFO, ECMO. *Paediatric Respiratory Reviews*.

[3] Eriksson J, Nordshus T, Carlsen KH (1986). Radiological findings in children with respiratory syncytial virus infection: relationship to clinical and bacteriological findings. *Pediatric Radiology*.

[4] Black CP (2003). Systematic review of the biology and medical management of respiratory syncytial virus infection. *Respiratory Care*.

[5] Rakshi I, Couriel JM (1994). Management of acute bronchiolitis. *Archives of Disease in Childhood*.

[6] Pezzotti P, Mantovani J, Benincori N, Mucchino E, Di Lallo D (2009). Incidence and risk factors of hospitalization for bronchiolitis in preterm children: a retrospective longitudinal study in Italy. *BMC Pediatrics*.

[7] Fodha I, Vabret A, Ghedira L (2007). Respiratory syncytial virus infections in hospitalized infants: association between viral load, virus subgroup, and disease severity. *Journal of Medical Virology*.

[8] Joffe S, Escobar GJ, Black SB, Armstrong MA, Lieu TA (1999). Rehospitalization for respiratory syncytial virus among premature infants. *Pediatrics*.

[9] Hall CB (1999). Respiratory syncytial virus: a continuing culprit and conundrum. *Journal of Pediatrics*.

[10] Wang EEL, Law BJ, Stephens D (1995). Pediatric investigators collaborative network on infections in Canada (PICNIC) prospective study of risk factors and outcomes in patients hospitalized with respiratory syncytial viral lower respiratory tract infection. *Journal of Pediatrics*.

[11] Meert K, Heidemann S, Abella B, Sarnaik A (1990). Does prematurity alter the course of respiratory syncytial virus infection?. *Critical Care Medicine*.

[12] Kern S, Uhl M, Berner R, Schwoerer T, Langer M (2001). Respiratory syncytial virus infection of the lower respiratory tract: radiological findings in 108 children. *European Radiology*.

[13] Friis B, Eiken M, Hornsleth A, Jensen A (1990). Chest X-ray appearances in pneumonia and bronchiolitis. Correlation to virological diagnosis and secretory bacterial findings. *Acta Paediatrica Scandinavica*.

[14] Kneyber MCJ, Moons KGM, De Groot R, Moll HA (2001). Predictors of a normal chest x-ray in respiratory syncytial virus infection. *Pediatric Pulmonology*.

[15] Papoff P, Moretti C, Cangiano G (2011). Incidence and predisposing factors for severe disease in previously healthy term infants experiencing their first episode of bronchiolitis. *Acta Paediatrica*.

[16] Prodhan P, Westra SJ, Lin J, Karni-Sharoor S, Regan S, Noviski N (2009). Chest radiological patterns predict the duration of mechanical ventilation in children with RSV infection. *Pediatric Radiology*.

[17] Tasker RC, Gordon I, Kiff K (2000). Time course of severe respiratory syncytial virus infection in mechanically ventilated infants. *Acta Paediatrica, International Journal of Paediatrics*.

[18] Jobe AH, Bancalari E (2001). NICHD/ NHLBI/ORD Workshop summary—Bronchopulmonary dysplasia. *American Journal of Respiratory and Critical Care Medicine*.

[19] American Academy of Pediatrics Committee on Infectious Diseases and Committee on Fetus and Newborn (2003). Revised indications for the use of palivizumab and respiratory syncytial virus immune globulin intravenous for the prevention of respiratory syncytial virus infections. *Pediatrics*.

